# Cardiovascular Diseases among Suiciders: A Population-Based Study in Northern Finland Population

**DOI:** 10.1155/2010/302102

**Published:** 2010-07-13

**Authors:** Arja Mainio, Helinä Hakko, Pirkko Räsänen, Markku Timonen

**Affiliations:** ^1^Department of Psychiatry, University of Oulu, P.O. Box 5000, 90014 Oulu, Finland; ^2^Department of Psychiatry, Oulu University Hospital, P.O. Box 26, 90029 OYS, Finland

## Abstract

*Objective*. Depression has been found to be an independent risk factor with cardiovascular diseases (CVDs) and also associated with increased mortality among these patients. *Method*. We used a comprehensive database of all suicides (*n* = 2, 283) committed in Northern Finland with information on all hospital-treated cardiovascular diseases and psychiatric disorders. *Results*. Coronary artery disease (CAD) had been present in 7.7% and other cardiovascular diseases (CVDs) in 11.6% of the suiciders. The likelihood of suicide for patients with hospital-treated CAD was estimated to be two-fold compared to the general population while likelihood for suicide was not elevated among those with other CVDs. Males with CAD and females with CAD or any CVD had been hospitalized significantly more often with depression compared to reference group. *Conclusions*. Suicidality among patients with cardiovascular diseases has been suggested to associate with depression. Psychiatric consultation is highly recommended in clinical practice for cardiac patients with depression or alcohol-related disorders.

## 1. Introduction

Depression has shown to be an independent risk factor for the onset of various cardiovascular diseases (CVDs) [1–4], such as myocardial infarction, coronary artery disease and cerebrovascular diseases, reviewed by Van der Kooy, and his coworkers [3]. The background behind this association has not been fully solved, and it has been suggested that depression causes heart disease either through behavioral factors, autonomic dysfunction, or inflammatory intermediates [5]. Furthermore, both major depression and depressive symptoms after myocardial infarction are linked with increased mortality as an independent risk factor among cardiac patients [6, 7]. 

Although depression and CVD are found to be highly and reciprocally associated with each other, there are only a few studies on suicidality among cardiac patients [8, 9]. Given the high prevalence (12–20%) of depression in hospitalized cardiac patients [10] and the possibility that depression is a more important predictor of suicide in the hospital than in the community, diagnosing depression in hospital settings may be a major factor in suicide prevention [11]. 

In order to study suicides among cardiac patients we had an access to a large Finnish suicide database. The aims of the present study were, firstly, to study the prevalence of cardiovascular diseases (CVDs) in the suicide population, and secondly, to evaluate separately the specific features of suicide completers according to CVD; that is, either coronary artery disease (CAD) or other CVDs. Further, we also calculated an estimate for the probability of suicide for patients with hospital-treated CAD as well as other CVDs in the general population.

## 2. Methods

We examined all suicides (*n* = 2,283) committed during the years 1988–2007 in the province of Oulu in Northern Finland. The data on age, gender, suicide methods, previous suicide attempts, and abuse of alcohol at the time of the suicide were based on death certificates from forensic medical-legal investigations. The Ethics Committee of Oulu University has approved the study protocol. The diagnoses of subjects were extracted from the Finnish Hospital Discharge Registers (FHDR). The FHDR covers all treatment episodes in general, mental, military, prison, and private hospitals, as well as the inpatient wards of local health centers nation-wide. The FHDR contains the personal and hospital identification codes, data on age, gender, length of stay, and primary diagnosis at discharge, together with three subsidiary diagnoses. The International Classification of Diseases (ICDs) was used as follows: CVD were classified as (1) Coronary artery disease (CAD) ICD8: 410–414.99 and 440–441.99; ICD9: 410–4149X and 440–4409X; ICD10: I20–I25 and I70–I70.9 and (2) Other cardiovascular diseases ICD8: 390–398.99, 400–404.99, 420–429.99 and 441–448.09; ICD9: 390–3989X. 401–4059X, 420–4299X and 441–4489X; ICD10: I00–I02, I05–I15, I30–I52, I71–I74, I77–I79.8. 

Psychiatric disorders were categorized as follows: (1) Depression ICD-8: 2960, 2980, 3004; ICD-9: 2961, 2968, 3004; ICD-10: F32-F34.1. (2) Psychotic disorders ICD-8: 296–299; ICD-9: 2961-4E, 2967A, 297, 298, 2990, 2999; ICD-10: F22-25, F28, F29, F30.2, F31.2, F31.5, F32.3, F33.3, (3) Substance use disorders ICD-8 303-304; ICD-9 303-305; ICD-10:F10-19. (4) Other psychiatric disorders; all other diagnoses apart early mentioned. We used in the present study primary diagnosis as well as subsidiary diagnoses.

The prevalence of suicides, CVDs, and population figures for those aged 15 years or older were obtained from Statistics Finland and the database from the National Institute of Health and Welfare.

The methods for suicide among study subjects were categorized as violent if the method was hanging, drowning, shooting, wrist cutting, jumping from a height, or traffic. Nonviolent methods were poisoning, gas, or other methods. Acute alcohol intoxication was detected in the medical autopsy by a doctor in forensic medicine and entered on the death certificate.

## 3. Statistical Analyses

Statistical significance of group differences in categorical variables was examined with Pearson *χ* ^2^-test or Fisher Exact test. Statistical significance of group differences in continuous variables was analyzed with the Mann-Whitney U-test or Kruskall-Walls test. The statistical significance of differences between repeated measurements were assessed with Wilcoxon signed rank test (two repeated measurements). By using the prevalence of suicide and CVDs in the general Finnish population and prevalence of CVDs in our database Bayes Rule for conditional probabilities was used to calculate the probability of suicide given that the person had any CVD. All statistical analyses were performed by using the SPSS statistical software, version 17, and the results in this study were considered statistically significant when the appropriately calculated two-tailed *P*-value was ≤ .05.

## 4. Results

Of the total suicide data population (*n* = 2,283), 19.3% (*n* = 442) had hospital-treated any CVD during their lifetime. CAD had been diagnosed in 7.7% (176) and other CVD in 11.6% (266) of the suiciders. Table 1 shows the clinical and suicide characteristics of the suiciders with or without CVD. Compared to other suiciders, those with other CVD than CAD had used significantly more often a nonviolent method, *P* = .050 Pearson *χ* ^2^-test. The most commonly used suicide method among suiciders with CAD (36.4%) and CVD (34.6%) was hanging, followed by shooting and poisoning among CAD sufferers, while those with other CVD than CAD had used poisoning. The majority of the suiciders with CVD (65%) were not under the influence of alcohol at the time of suicide. 

Table 2 shows hospital-treated psychiatric disorders according to gender. The suiciders with CVDs had significantly more often hospital-treated psychiatric disorders in their life-time history compared to those with no CVD. Both the male and female suiciders with CAD had significantly more often been treated in hospital due to their depression compared those with no CVD. Females with other CVDs had hospitalized significantly more often due their depression than those without any CVD. The male suiciders with any CVD had significantly more often alcohol-related disorders compared to those without any CVD. 

The Bayes Rule for conditional probabilities was used to estimate the probability of suicide among hospital-treated patients with CVDs. The prevalence of suicide in Finnish general population is 0.03%. The prevalence of hospital-treated CADs, as well other CVDs in the general Finnish population (aged 15 years or over) are 3.7%, and 10.8%, respectively, (Table 3). In our study, the prevalence of CAD among suicide suiciders was 7.7% and other CVDs 11.6%, respectively. The probability of suicide among patients with CAD was 0.06% and among suiciders with other CVDs was 0.03%. Thus, the likelihood of suicide was two-fold for patients with hospital-treated CADs compared to the general population, while likelihood for suicide was among those with other CVDs was the same as likelihood for suicide in the general population.

## 5. Discussion

We found that about one in five of all the subjects in this large population-based suicide data set from Northern Finland had suffered from any cardiovascular disease. To our knowledge, this is the first time that life-time prevalence of CVDs was investigated among suiciders. Earlier studies have pointed out an increase in suicide attempts among CAD patients [8]. In the present study, about 8% of the suiciders had CAD and 12% from other CVDs than CAD. The prevalence of hospital-treated CVDs among suiciders was not elevated being about the same as in general population in Finland which is shown to be 11%. However, the prevalence of hospital-treated CAD among suiciders was twice as high as in general population [12]. In terms of the probability of suicide among patients with hospital-treated CAD, these patients were estimated to have a two-fold risk for suicide than in the general population. Our finding is in line with the Brazil mortality study which recently found that the incidence of deaths from suicide was positively correlated with deaths from ischemic heart disease but not from other cardiovascular diseases [11]. Also, it has previously been shown that the percentage of suicides in persons with cerebrovascular disease, that is, among stroke patients was as high as 7.2% in the Danish population [13]. Further studies would be needed to extract more information on special risk factors for suicide risk among CAD patients.

Cardiovascular diseases, especially CAD, are often associated with depression [1–3]. In our study, both the male and female suiciders with CAD had been diagnosed with depression more often than the reference ones. The prevalence of hospital treated depression in Finnish population according to Finnish Hospital Discharge Registers (FHDR) [12] has shown to be about the same as in the suiciders with CVDs in the present study. However, the prevalence of depression among suiciders with CAD was twice as the corresponding prevalence in Finnish hospital patients. We suggest that depression among CAD patients is a remarkable contributor for suicide. 

The prevalence of substance-related diseases was also higher in the males with any cardiovascular disease but this prevalence was not higher than among the patients with Finnish general hospital treated patients. Also, alcohol was significantly less often contributed to suicide among those with any cardiovascular diseases compared to those without CVDs. In our opinion, at least two issues in the background of this result can be noticed. Firstly, we suggest that suicide is mainly committed without alcohol intake and is thus nonimpulsive and premeditated before the definitive accomplishment of suicide. Secondly, the lower alcohol influence can be associated with significantly older age among CVD suiciders. 

According to our study, the profile of a typical suicider with CAD can be described as follows: an elderly male hospitalized for depression or alcohol-related disease. The method for suicide is likely to be violent (mostly hanging), and alcohol will not contribute to the suicide. Heavy alcohol abuse has been shown to be related to increased risk of cardiac diseases. In addition, alcohol dependence is also in general highly associated with risk of suicide [14, 15]. Psychiatric consultation is highly recommended in clinical practice for cardiac patients with depression or alcohol-related disorders. It is well known that recognition of depression and treatment with antidepressive drugs are the most important factors in suicide prevention [16]. 

The strength of this study is that all the suicides committed in the geographically large area of Northern Finland during a 20-year period were included and evaluated. Thus, the series was not restricted to a certain age group, nor were the suicide data otherwise limited in any way. In addition, the source for the diagnoses attached to the hospital treatments was the Finnish Hospital Discharge Register, which has been shown to be reliable for use in scientific research [17]. Similarly, the Finnish death certificate form, the certification practices involved and the system for determining the cause of death have all been shown to possess acceptable validity for mortality research [18]. 

Since our data did not enable us to calculate the standardized mortality ratio for expected deaths, we used the Bayes' rule for conditional probabilities of suicide and CVDs. Other limitation of our study was that the ICD diagnoses extracted from the Finnish Hospital Discharge Register only include patients who have been admitted to hospital. This means that only severe cases of cardiovascular diseases that needed hospitalization are included. Unfortunately, there is no national register of outpatient treatments in Finland, and therefore our reference group may include some false-negative cases with respect to both cardiovascular diseases and depression. Also, the causality between cardiovascular diseases and depression could not be evaluated.

## Figures and Tables

**Table 1:** The characteristics of the suicide victims according to cardiovascular disease.

	Coronary artery disease *n*(%) = 176	Other cardiac disease *n*(%) = 266	No cardiac disease *n*(%) = 1841	*P*-value*
Males	156 (88.6)	191 (71.8)	1516 (82.3)	<.001

Mean (std) age in years at the time of death	62.1 (12.9)	52.7 (14.3)	40.3 (14.4)	<.001**

Violent method for suicide	129 (73.3)	183 (68.8)	1393 (75.7)	.050

Hanging	64 (36.4)	92 (34.6)	512 (27.8)	<.001
Shooting	38 (21.6)	54 (20.3)	569 (30.9)	
Poisoning	37 (21.0)	73 (27.4)	322 (17.5)	
Gas	10 (5.7)	10 (3.8)	126 (6.8)	
Traffic	4 (2.3)	2 (0.8)	87 (4.7)	
Jumping	5 (2.8)	5 (1.9)	44 (2.4)	
Drowning	14 (8.0)	23 (8.6)	125 (6.8)	
Other	4 (2.3)	7 (2.6)	56 (3.0)	

Previous suicide attempt	14 (8)	37 (13.9)	193 (10.5)	.547

Alcohol contributed to the suicide	62 (35.2)	92 (34.6)	849 (46.1)	<.001

*Pearson *χ* ^2^-test or Fisher Exact test when appropriate, two-tailed significance.

**Kruskall-Wallis test, two-tailed significance.

**Table 2:** Prevalence of psychiatric disorders among suiciders in males and females.

	Males				Females			
Psychiatric disorder	Coronary artery disease *n*(%) = 156	Other cardiac disease *n*(%) = 191	No cardiac disease *n*(%) = 1516	*P* value*	Coronary artery disease *n*(%) = 20	Other cardiac disease *n*(%) = 75	No cardiac disease *n*(%) = 325	*P* value*
Depression	43 (27.6)	29 (15.2)	164 (10.8)	< .001 .005**	7 (35.0)	35 (46.7)	82 (25.2)	.001
Alcohol-related disorders	34 (21.8)	47 (24.6)	208 (13.7)	< .001	2 (10.0)	4 (5.3)	26 (8.0)	.676

*Fischer exact test, two-tailed significance.

**Coronary artery disease versus other cardiovascular disease.

**Table:** (a) Numbers of patients with hospital-treated cardiovascular diseases in general population according to Finnish Hospital Discharge Registers (FHDR).

	Coronary artery disease	Other cardiovascular disease	No cardiovascular disease	Total
Patients	30579 (3.7%)	90255 (10.8%)	717578 (85.5%)	838412
Males	56%	48%	43%	
Mean age in yrs	75	70	64	

**Table:** (b) Number of hospital-treated patients according to psychiatric disorders.

	Depression	Alcohol related disorders	Any psychiatric disorder
Patients	9951 (15%)	15738 (24%)	64238
Males	38%	78%	51%
Age in yrs	54	48	50
